# Comparing the effect of positioning on cerebral autoregulation during radical prostatectomy: a prospective observational study

**DOI:** 10.1007/s10877-020-00549-0

**Published:** 2020-06-20

**Authors:** Stefanie Beck, Haissam Ragab, Dennis Hoop, Aurélie Meßner-Schmitt, Cornelius Rademacher, Ursula Kahl, Franziska von Breunig, Alexander Haese, Markus Graefen, Christian Zöllner, Marlene Fischer

**Affiliations:** 1 Department of Anesthesiology, University Medical Center Hamburg-Eppendorf, Martinistraße 52, 20246 Hamburg, Germany; 2 Martini-Klinik, Prostate Cancer Center, University Medical Center Hamburg-Eppendorf, Hamburg, Germany; 3 Department of Intensive Care Medicine, University Medical Center Hamburg-Eppendorf, Hamburg, Germany

**Keywords:** Anesthesia, Autoregulation, Cerebral blood flow, Head-down tilt, Prostatectomy, Supine position

## Abstract

**Purpose:**

Surgery in the prolonged extreme Trendelenburg position may lead to elevated intracranial pressure and compromise cerebral hemodynamic regulation. We hypothesized that robot-assisted radical prostatectomy with head-down tilt causes impairment of cerebral autoregulation compared with open retropubic radical prostatectomy in the supine position.

**Methods:**

Patients scheduled for elective radical prostatectomy were included at a tertiary care prostate cancer clinic. Continuous monitoring of the cerebral autoregulation was performed using the correlation method. Based on measurements of cerebral oxygenation with near-infrared spectroscopy and invasive mean arterial blood pressure (MAP), a moving correlation coefficient was calculated to obtain the cerebral oxygenation index as an indicator of cerebral autoregulation. Cerebral autoregulation was measured continuously from induction until recovery from anesthesia.

**Results:**

There was no significant difference in cerebral autoregulation between robot-assisted and open retropubic radical prostatectomy during induction (p = 0.089), intraoperatively (p = 0.162), and during recovery from anesthesia (p = 0.620). Age (B = 0.311 [95% CI 0.039; 0.583], p = 0.025) and a higher difference between baseline MAP and intraoperative MAP (B = 0.200 [95% CI 0.073; 0.327], p = 0.002) were associated with impaired cerebral autoregulation, whereas surgical technique was not (B = 3.339 [95% CI  1.275; 7.952], p = 0.155).

**Conclusion:**

Compared with open radical prostatectomy in the supine position, robot-assisted surgery in the extreme Trendelenburg position with capnoperitoneum did not lead to an impairment of cerebral autoregulation during the perioperative period in our study population.

**Trial registration number**: DRKS00010014, date of registration: 21.03.2016, retrospectively registered.

**Electronic supplementary material:**

The online version of this article (10.1007/s10877-020-00549-0) contains supplementary material, which is available to authorized users.

## Introduction

Radical prostatectomy is one curative treatment option for localized prostate cancer in patients with a life expectancy greater than 10 years [1]. With the increasing adoption of minimally invasive techniques, robot-assisted radical prostatectomy (RARP) has become the predominant surgical approach for radical prostatectomy [2]. Compared with open retropubic surgery, the advantages of RARP include less blood loss and lower transfusion rates [3–5]. Moreover, RARP has been shown to enhance postoperative recovery and reduce hospital length of stay [3, 4]. Independently from these beneficial effects, however, RARP has not been shown to be superior in terms of urological and oncological outcome or quality of life [6–10]. Notably, it is under debate, whether reduced health-economic expenses and benefits in quality-adjusted life years outweigh the high costs associated with robot-assisted surgery [11, 12].

To allow for visual inspection of the surgical field, RARP requires peritoneal insufflation of carbon dioxide and the extreme Trendelenburg position with a 45-degree head-down tilt [13]. In contrast with RARP, open retropubic radical prostatectomy (ORP) is performed in the supine position. Although the head-down tilt with capnoperitoneum is well tolerated by the majority of patients, there are concerns about the effects on several organ systems, including cardiovascular and pulmonary function as well as cerebrovascular hemodynamic regulation [14, 15]. As a result of increased venous return and the effect of gravity in the head-down position, elevated intracranial pressure has been observed [16]. Moreover, observational studies report an increase in intraocular pressure and optic nerve sheath diameter in the steep Trendelenburg position compared with supine positioning [17–19]. Resulting from elevated intraocular pressure visual loss due to ischemic optic neuropathy has been reported in single cases [20, 21]. Elevated intracranial pressure during head-down position may affect cerebral perfusion [22].

Cerebral blood flow is tightly regulated to ensure constant cerebral perfusion by autoregulation of the cerebral circulation [23]. Vasoactive mediators induce vasodilation in response to hypotension to avoid cerebral hypoperfusion with the risk of ischemia or trigger cerebral vasoconstriction following hypertension to prevent cerebral hyperperfusion [24]. There are conflicting results on the influence of the steep Trendelenburg position in combination with pneumoperitoneum on the cerebral vasculature [17, 25, 26]. Previous studies have not compared perioperative changes of cerebral autoregulation between head-down and supine position, but assessed cerebral autoregulation during Trendelenburg position only. We hypothesized that cerebrovascular autoregulation might be impaired in patients undergoing RARP in the head-down position compared with patients scheduled for ORP in the supine position. Therefore, we performed continuous perioperative measurement of the cerebral autoregulation in patients undergoing elective radical prostatectomy for prostate cancer with the robot-assisted or the open surgical technique.

## Methods

### Ethics, setting, and study design

This study was approved by the local Ethics Committee at Hamburg Medical Chamber (PV4782). All patients gave written informed consent before study participation. The study was performed in accordance with the 1964 Declaration of Helsinki and its later amendments. This prospective observational study was performed at a tertiary care prostate cancer clinic between June 2015 and March 2016. The choice for open versus robot-assisted surgery was based on surgical (i.e., prostate size and prior abdominal surgery) and oncological considerations, including Gleason score and T stage, patient-related risk factors, such as stenotic valvular disease or obesity, and patient preference.

### Participants

Patients were chosen by convenience and were included if they were scheduled for elective radical prostatectomy for prostate cancer and were fluent in German in order to understand the informed consent form. We did not include patients with a history of cerebrovascular or neurodegenerative disease or any other intracranial pathology. The Mini-Mental Status Examination was used to identify patients with mild cognitive impairment [27]. Patients in whom invasive blood pressure monitoring in the radial arteries was not feasible, for example because of severe atherosclerosis, were excluded from the study.

### Blood pressure measurement

Before anesthesia induction, three blood pressure values were obtained by repeated oscillometric measurement in three-minute intervals with the patient in the supine position. The baseline blood pressure was calculated as the mean of these three initial measurements.

Invasive blood pressure monitoring was performed in all patients from the beginning of anesthesia induction and was continued for one hour after arrival in the post-anesthesia care unit (PACU) or until the patient was considered hemodynamically and respiratory stable for transfer to the normal ward. Under aseptic conditions, a 20G arterial cannula (BM™, BD Flowswitch™, BD Germany) was inserted in the left radial artery after local anesthesia with plain lidocaine 1%. If placement of the cannula was not feasible in the left radial artery, the right radial artery was chosen for invasive blood pressure measurement. The arterial cannula was connected to tubing filled with saline that transmits a pressure wave to a transducer and a flushing system consisting of 500 ml saline pressurized to 300 mmHg. The transducer was placed at the level of the right atrium.

### Monitoring of cerebrovascular autoregulation

Cerebral autoregulation was measured continuously using the time correlation method, which has been described in detail previously [28]. This approach is based on continuous monitoring of the mean arterial blood pressure (MAP) and a cerebral blood flow surrogate. For this trial, we used non-invasive monitoring of the cerebral oxygenation with near-infrared spectroscopy (INVOS™ 5100 Cerebral Oximeter, Medtronic GmbH, Meerbusch, Germany) as a surrogate for cerebral blood flow. A moving linear correlation between MAP and cerebral oxygenation was calculated based on a sliding 300-s window updated every 10 s (ICM + , Cambridge Enterprise Ltd, Cambridge, UK). This method provides the cerebral oxygenation index (COx), which has been shown to reliably mirror cerebral autoregulation [29, 30]. A COx close to zero indicates no correlation between MAP and cerebral oxygenation and reflects cerebral blood flow within the autoregulatory range. By contrast, a more positive correlation is indicative for an impaired cerebrovascular autoregulation. COx levels > 0.3 are regarded as an indicator of a pathological cerebral autoregulatory response to systemic blood pressure fluctuations [31].

### Anesthetic procedures

General anesthesia was induced using sufentanil 0.3–0.7 µg/kg, propofol 2–3 mg/kg and rocuronium 0.5–0.6 mg/kg. Anesthesia was maintained using sevoflurane at MAC 0.8–1.2 in combination with sufentanil 0.1–0.2 µg/kg. Anesthesia depth was monitored with the bispectral index.

Muscle relaxation was performed intermittently with rocuronium 0.15 mg/kg when the anesthesiologist deemed it necessary. Intraoperative hypotension (MAP < 65 mmHg or a decrease in MAP > 20% of baseline for more than 5 min) was managed with a bolus application of norepinephrine 5–10 µg followed by an incremental administration of continuous norepinephrine infusion. End-tidal carbon dioxide (CO_2_) was continuously monitored and maintained between 32 and 42 mmHg.

### Positioning

Patients undergoing ORP remained in the supine horizontal position throughout the surgical procedure. In patients who had RARP, the head-down position was established after peritoneal insufflation of CO_2_. The steep Trendelenburg position was maintained with a 45-degree head-down tilt. Intraabdominal pressure was measured continuously and was maintained at 10 mmHg.

### Statistical analysis

Mean arterial blood pressure and COx values were obtained in one-minute intervals. Mean MAP and mean COx were calculated for predefined time periods of interest: (1) from bolus administration of sufentanil for anesthesia induction until the patient was transferred from the anesthesia induction room to the operating theater (induction), (2) from incision to closure (intraoperative), (3) from incision until the start of capnoperitoneum (before capno, RARP patients only), (4) 20 min from the start of capnoperitoneum (capno start, RARP patients only), (5) during the Trendelenburg position (RARP patients only), (6) 10 min following bolus application of norepinephrine, (7) during the first 60 min of PACU stay and (8) throughout the entire monitoring period (from the start of anesthesia induction until 60 min after arrival in the PACU). Variability of MAP and COx were calculated as the variance of all MAP/COx values throughout the measurement period. Impaired cerebral autoregulation was defined as a COx > 0.3. The percentage of the entire monitoring period with the COx > 0.3 was calculated for each patient and defined as primary endpoint.

Data are presented as median (interquartile range) or n (%) unless stated otherwise. Continuous variables were compared between groups with the Mann–Whitney U test. For categorical variables, the Chi-square test or the Fisher’s exact test was used as appropriate. Mean COx levels during different perioperative episodes (induction, intraoperative, and PACU for ORP; induction, before capno, head-down, and PACU for RARP) were compared with the Wilcoxon signed-rank test and Bonferroni corrected for multiple comparisons.

To determine the influence of surgical technique on cerebral autoregulation, an analysis of covariance was performed with the time with COx > 0.3 in % as the dependent variable. Augmented stepwise backward elimination was used to identify factors associated with impaired autoregulation. Surgical technique and clinically relevant parameters that did not fulfill the criteria for collinearity were included as fixed factors (categorical variables: surgical technique [robot-assisted vs. open surgery], cardiovascular risk factors [0–1 vs. ≥ 2], high vasopressor support,) or covariates (continuous variables: age, mini-mental status examination score, duration of surgery, estimated blood loss, MAP variability throughout the perioperative period, difference between pre-induction and intraoperative MAP). Subsequently, variables that were significant on a 0.2 level were eliminated to create a preliminary model. Using a stepwise approach eliminated variables were included in the preliminary model and checked for a change in parameter estimate (B for surgical technique) of > 10%.

All data were analyzed with SPSS 22 (IBM SPSS® Statistics, IBM Corporation). Figures were designed with GraphPad Prism 8.2 (GraphPad Software Inc., San Diego, California).

## Results

**Fig. 1:**
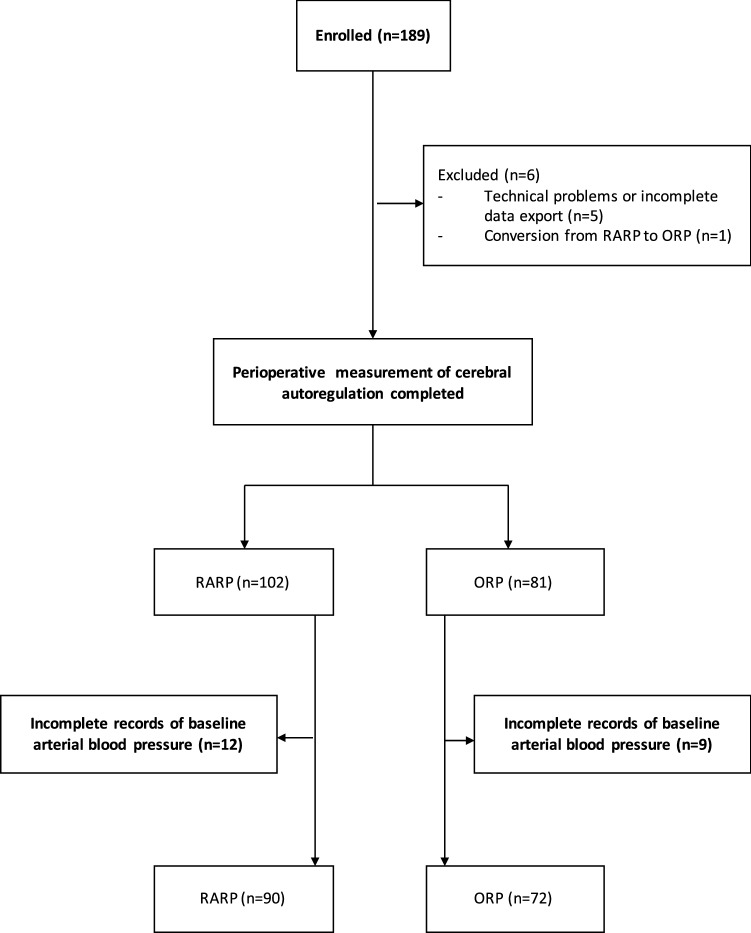
Flow of study participants throughout the study

A total of 189 patients were enrolled. Data on cerebral autoregulation were available from 183 patients. Of these, 102 (55.7%) patients underwent RARP in the steep Trendelenburg position, and 81 (44.3%) patients had ORP in the supine position. The flow of study participants is presented in Fig. 1. The median age of the study population was 63 years (range 45–76). Apart from dyslipoproteinemia and coronary heart disease, which was present more often in patients undergoing open surgery, the two surgical groups did not differ significantly with regard to comorbid conditions. For details on demographic and clinical characteristics of the study population, see Table 1. All patients fulfilled the criteria for categories 1 to 3 of the American Society of Anesthesiologists physical status classification system.

**Table 1:** Baseline characteristics and variables related to anesthesia and surgery

	RARP (n = 102)	ORP (n = 81)	*p*
Baseline demographic and clinical characteristics			
Age (years)	63 (58–67)	63 (60–68)	0.223
Body Mass Index	26.4 (24.3–29.4)	25.7 (24.5–28.4)	0.451
Obesity (BMI ≥ 30)	25 (24.5)	19 (23.5)	0.868
Arterial hypertension	53 (52.0)	41 (50.6)	0.857
Diabetes	5 (4.9)	5 (6.2)	0.752
Dyslipoproteinemia	22 (21.6)	29 (35.8)	0.033
Current smoking status	14 (13.7)	9 (11.1)	0.596
Coronary heart disease	6 (5.9)	14 (17.3)	0.014
Sleep apnea syndrome	7 (6.9)	2 (2.5)	0.302
Chronic obstructive pulmonary disease	5 (4.9)	5 (6.2)	0.752
Mini-mental status examination score	29 (28–30)	29 (27–29)	0.001
Anesthesia & surgery			
ASA Physical Status Classification System			0.094
I	16 (15.7)	21 (25.9)	
II	71 (69.6)	44 (54.3)	
III	15 (14.7)	16 (19.8)	
Total amount of fluids administered (ml)	2500 (2000–3000)	2500 (2000–3000)	0.257
Crystalloid fluids (ml)	2500 (2000–3000)	2500 (2000–2500)	0.017
Colloid fluids (ml)	0 (0–0)	500 (0–500)	< 0.001
High vasopressor support^a^	85 (83.3)	80 (98.8)	< 0.001
Duration of surgery (min)	193 (165–220)	165 (145–185)	< 0.001
Estimated blood loss (ml)	275 (200–400)	800 (600–1100)	< 0.001
Sufentanil (µg)	100 (90–120)	90 (80–100)	< 0.001

Categorical data are displayed as n (%), continuous data are presented as median (interquartile range)

^a^Continuous infusion of norepinephrine > 75% of surgical time. *ASA* American Society of Anesthesiologists

Estimated blood loss, requirement of fluid resuscitation, and vasopressor support were significantly higher during ORP (Table 1). Patients who had robot-assisted surgery received significantly higher doses of sufentanil and had longer procedures compared with those undergoing ORP. For urologic oncological features, see Online Resource 1.

### Blood pressure

The mean MAP during anesthesia induction did not differ significantly between groups. Mean MAP values from incision to closure, during the PACU stay, and throughout the monitoring period were significantly lower in ORP patients compared with RARP patients. Changes from baseline MAP to intraoperative MAP and from baseline MAP to PACU MAP differed significantly between surgical groups. For details on blood pressure levels, see Table 2.

**Table 2:** Average blood pressure levels during anesthesia induction, from incision to closure, during the first 60 min of PACU stay, and during the entire monitoring period

	RARP	ORP	*p*
Blood pressure from induction to recovery of anesthesia			
	n = 102	n = 81	
MAP (induction)	86 (78–94)	84 (76–89)	0.074
SBP (induction)	124 (113–133)	121 (115–133)	0.922
MAP (intraoperative)	83 (80–86)	76 (73–79)	< 0.001
SBP (intraoperative)	114 (110–120)	112 (107–118)	0.056
MAP variability (intraoperative)	90 (61–122)	81 (50–107)	0.030
MAP (PACU)	98 (89–107)	92 (83–103)	0.006
SBP (PACU)	156 (137–170)	149 (140–174)	0.730
MAP variability (PACU)	21 (13–47)	24 (11–44)	0.991
MAP (entire measurement period)	86 (83–89)	81 (76–85)	< 0.001
SBP (entire measurement period)	123 (117–129)	121 (116–127)	0.460
MAP variability (entire measurement period)	160 (113–202)	142 (93–208)	0.206
Mean arterial blood pressure before anesthesia induction and difference from baseline			
	n = 90	n = 72	
Baseline MAP	106 (100–115)	106 (100–115)	0.772
ΔMAP (baseline—induction)	20 (11–31)	24 (19–29)	0.06
ΔMAP (baseline—intraoperative)	24 (16–33)	31 (26–39)	< 0.001
ΔMAP (baseline—PACU)	11 (− 3to 23)	16 (7–24)	0.047

Baseline MAP was calculated as the average of three non-invasive blood pressure measurements prior to induction of anesthesia. ΔMAP was defined as the difference between mean induction, intraoperative, or PACU MAP from baseline. Data are presented as median (interquartile range)

*RARP* robot-assisted radical prostatectomy, *ORP* open retropubic radical prostatectomy, *MAP* mean arterial blood pressure, *SBP* systolic blood pressure, *PACU* post-anesthesia care unit

### Cerebrovascular autoregulation

There was no statistically significant association between surgical technique (RARP vs. ORP) and time with impaired autoregulation in the multivariable model (Table 3). Age and ΔMAP_baseline—intraoperative_ were associated with reduced cerebral autoregulatory function (Table 3).

**Table 3:** Multivariable analysis of covariance with the percentage of monitoring time with impaired cerebral autoregulation (COx > 0.3) as dependent variable

	B	95% CI	t	*p*
Surgical technique (RARP vs. ORP)	3.339	− 1.275;7.952	1.431	0.155
Vasopressor support^a^	− 5.038	– 10.715;0.639	− 1.755	0.082
Age (per year increase)	0.311	0.039;0.583	2.259	0.025
MMSE (per point increase)	0.778	− 0.212;1.768	1.554	0.122
Duration of surgery (per 30 min increase)	0.997	− 0.335;2.329	1.480	0.141
Estimated blood loss (per 100 ml increase)	0.315	− 0.028;0.659	1.816	0.072
ΔMAP_baseline—incision to closure_ (per mmHg increase)	0.200	0.073;0.327	3.121	0.002
Mean MAP_entire monitoring period_ (per mmHg decrease)	− 0.082	− 0.344;0.180	− 0.617	0.539

Augmented backward elimination of cardiovascular risk factors (0–1 vs. ≥ 2, p = 0.942) and variability of mean arterial blood pressure (MAP) throughout the entire monitoring period (p = 0.839)

^a^Continuous infusion of norepinephrine < 75% of surgical time vs. > 75%. *MMSE* Mini-mental status examination score, *RARP* robot-assisted radical prostatectomy, *ORP* open retropubic radical prostatectomy

**Fig. 2:**
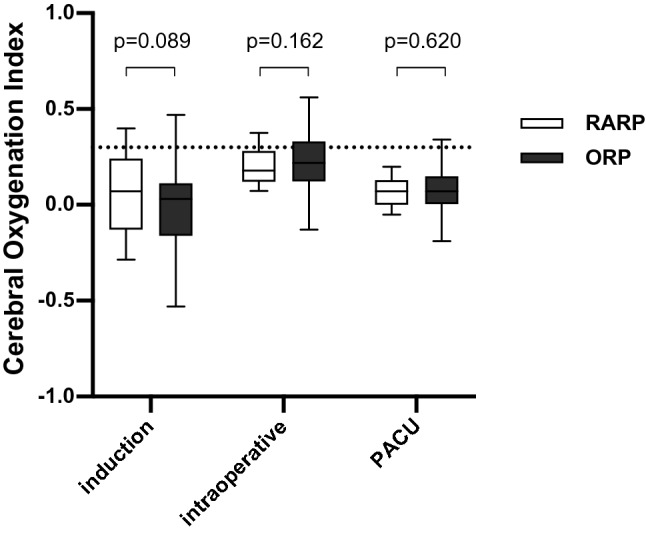
Cerebral oxygenation index as an indicator of cerebral autoregulation in patients with robot-assisted (RARP) and open retropubic radical prostatectomy (ORP) during anesthesia induction, intraoperatively from incision to closure, and during recovery from anesthesia in the post-anesthesia care unit (PACU). A cerebral oxygenation index above 0.3 (dotted line) denotes an impairment of cerebral autoregulation. Data are presented as median (horizontal line) with Tukey whiskers

**Fig. 3:**
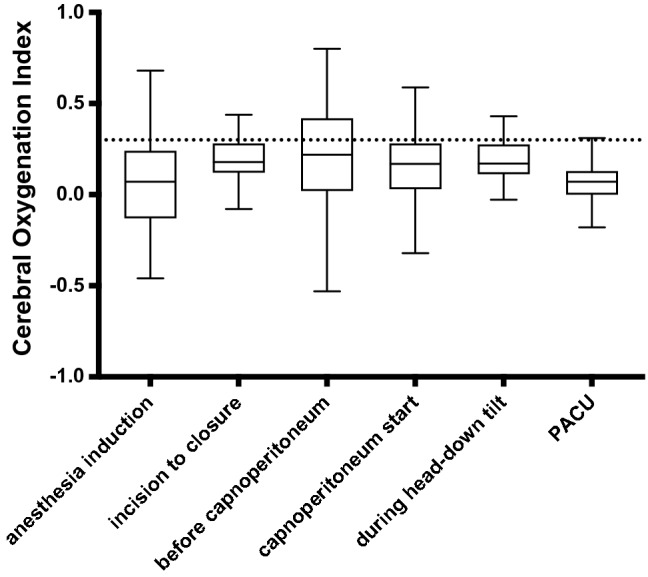
Median cerebral oxygenation index (indicating cerebral autoregulation) in patients, who underwent robot-assisted radical prostatectomy during different episodes throughout the perioperative period. A cerebral oxygenation index above 0.3 (dotted line) denotes an impairment of cerebral autoregulation. PACU: post-anesthesia care unit. Data are presented as median (horizontal line) with Tukey whiskers

We did not find a significant difference in mean COx values during anesthesia induction, during the intraoperative period from incision to closure, during the PACU stay or during the entire measurement period between RARP and ORP (data are displayed in Table 4 and Fig. 2). Cerebral autoregulation did not differ significantly between surgical groups following bolus application of norepinephrine (Table 4). In the RARP group, the mean COx was calculated before the onset of capnoperitoneum, during the first 20-min period after peritoneal insufflation of CO_2_ and during the head-down position (Fig. 3). Mean COx was below the pathologic threshold of 0.3 during each of these episodes, indicating preserved cerebral autoregulation. COx levels before capnoperitoneum and during head-down tilt were significantly higher compared with COx during anesthesia induction and during the PACU stay in patients undergoing RARP. In RARP patients, COx did not differ significantly between induction and PACU stay.

**Table 4:** Average cerebral oxygenation index (COx, minimum − 1 to maximum + 1) during various perioperative episodes, beginning from induction of anesthesia until the first 60 min of the post-anesthesia care unit (PACU) stay

	RARP (n = 102)	ORP (n = 81)	*p*
COx (induction)	0.07 (− 0.13;0.24)	0.03 (− 0.16;0.11)	0.089
COx (intraoperative)	0.18 (0.12;0.28)	0.22 (0.12;0.33)	0.162
COx (PACU)	0.07 (0.00;0.13)	0.07 (0.02;0.15)	0.620
COx (after norepinephrine bolus)	0.14 (− 0.04;0.29)	0.14 (0.02;0.28)	0.517
COx (entire measurement period)	0.15 (0.09;0.22)	0.17 (0.09;0.25)	0.537
COx (before capnoperitoneum)	0.22 (0.02;0.42)	–	–
COx (20 min from abdominal CO_2_ insufflation)	0.17 (0.03;0.28)	–	–
COx (during head-down position)	0.17 (0.11;0.27)	–	–
COx variability	0.16 (0.14;0.19)	0.16 (0.13;0.19)	0.345
COx > 0.3 (% of monitoring time)	38.1 (31.8;44.4)	40.1 (32.4;46.8)	0.253

Impaired cerebral autoregulation was defined as COx > 0.3. Data are presented as median (interquartile range)

*RARP* robot-assisted radical prostatectomy, *ORP* open retropubic radical prostatectomy

In the ORP group, intraoperative COx was significantly higher than COx during anesthesia induction and COx during the PACU stay. Postoperative COx during PACU was significantly lower compared with induction COx in ORP patients. For comparisons of COx between episodes, see Online Resource 2.

### Subgroup analysis

About one-half of the study population (n = 94/183, 51.4%) had a history of arterial hypertension. We compared COx levels during anesthesia induction, from incision to closure, during the PACU stay, and after bolus administration of norepinephrine between patients with and without arterial hypertension. In the RARP group, COx levels before capnoperitoneum, 20 min after the beginning of capnoperitoneum and in the head-down position were compared between patients with and without arterial hypertension. There was no significant difference in COx levels during any of these episodes (Online Resource 3a). Eighty-eight patients (54.0%) for whom baseline MAP values were available suffered from arterial hypertension. In these patients, baseline MAP was significantly higher (109 mmHg [IQR 101–116]) than in patients without arterial hypertension (104 mmHg [IQR 96–114], p = 0.040). Accordingly, ΔMAP_baseline – induction_ (24 mmHg [IQR 16–31] vs. 21 mmHg [IQR 14–27], p = 0.035) and ΔMAP_baseline – intraoperative_ (29 mmHg [IQR 21–39] vs. 25 mmHg [IQR 14–33], p = 0.013) differed significantly between patients with and without arterial hypertension.

When analyzing COx in hypertensive patients only, we did not find a significant difference between RARP and ORP during any perioperative episode (Online Resource 3b).

## Discussion

The aim of this prospective observational study was to compare continuous analyses of cerebrovascular autoregulation between RARP in the Trendelenburg position and ORP in the supine position. (1) We found that cerebral autoregulation did not differ between robot-assisted and open surgery during the perioperative period. (2) Increasing age and a high difference between pre-induction and intraoperative MAP were associated with impaired cerebral autoregulation. (3) Compared with anesthesia induction and recovery from anesthesia, intraoperative cerebral autoregulatory function was reduced in both surgical groups independently from positioning.

Concern about the steep Trendelenburg position with capnoperitoneum is in part attributable to case reports of postoperative visual loss because of ischemic optic neuropathy [20, 21, 32]. Several trials have reported on the effects of the Trendelenburg position on intraocular and intracranial pressure as a result of decreased return of cerebral venous blood [16, 17, 19, 33, 34].

Elevated intracranial pressure during Trendelenburg position may compromise cerebral perfusion and the regulation of cerebral blood flow [22]. Interestingly, we did not find a difference in cerebral autoregulation between RARP in the head-down tilt and ORP in supine position. Our results are in contrast to findings from a previous trial. Schramm and colleagues observed impaired cerebrovascular autoregulation during RARP in the Trendelenburg position [26]. In a prospective observational study, they measured cerebral autoregulation using transcranial Doppler sonography in 23 patients. They found decreasing function of the cerebral autoregulation corresponding to the duration of extreme positioning. Conversely, after repositioning a return to baseline levels of cerebral autoregulatory function was observed. This effect is in line with our results of decreased intraoperative cerebral autoregulation in RARP patients. However, in contrast with Schramm et al. we included patients undergoing ORP in addition to RARP patients and found similar patterns of perioperative changes of cerebral autoregulation in both surgical groups.

Under physiological conditions, adequate cerebral blood flow is preserved independently from systemic blood pressure, if cerebral perfusion pressure is between 50 and 150 mmHg, referred to as the lower and upper limits of cerebral autoregulation [28]. If systemic blood pressure is higher or lower than the autoregulatory limits, cerebral blood flow becomes more dependent on perfusion pressure, ultimately resulting in a linear flow-pressure relationship [35]. These cerebral autoregulatory limits are not fixed and may be subject to interindividual and intraindividual variation depending on age or comorbid conditions [36, 37]. One well-studied example is the right shift of the autoregulatory curve in individuals with arterial hypertension, rendering cerebral blood flow more susceptible to low systemic blood pressure [38].

In the present study, arterial hypertension was not associated with prolonged perioperative impairment of the cerebral autoregulation. However, patients with a history of arterial hypertension had more elevated pre-induction blood pressure levels, and the difference between pre-induction MAP and intraoperative MAP was significantly associated with the duration of impaired autoregulation.

We found increasing age to be associated with impaired cerebral autoregulation during the perioperative period in patients undergoing radical prostatectomy. This effect was present regardless of surgical technique. There are conflicting results regarding age-dependent alterations in cerebrovascular autoregulation. Inducing changes of MAP and arterial CO_2_, Oudegeest-Sander and colleagues showed that cerebral autoregulation and reactivity to CO_2_ were preserved in healthy elderly individuals [39]. Interestingly, in comparison with young patients, no clinically relevant difference in the regulation of cerebral blood flow has been observed in patients older than 65 years during general anesthesia [40]. Conversely, cerebral perfusion has been shown to be differentially regulated after posture change in elderly patients compared with young participants [41]. Intraocular pressure and optic nerve sheath diameter, as a surrogate for intracranial pressure, were monitored in 51 patients undergoing RARP in the head-down position [17]. Compared with younger participants, elderly patients had reduced compliance for the steep head-down tilt with impaired adaptation to prolonged positioning.

In addition to patient-related factors such as age and hypertension, extrinsic factors may affect cerebral autoregulation. Anesthetics may have an impact on the cerebrovascular tone, alter autoregulatory limits and narrow the autoregulatory plateau [42]. Lower limits of autoregulation during inhalational anesthesia with sevoflurane have been reported to be around 73 mmHg (± 14) in patients older than 65 years [36]. Individual variation of autoregulatory limits may compromise cerebral perfusion at blood pressure levels, which would traditionally be considered sufficient for organ perfusion. These findings suggest that lower autoregulatory limits of 90 mmHg might occur in some patients, who would require blood pressure targets substantially higher than 65 mmHg to maintain cerebral autoregulatory function during general anesthesia.

We found decreased cerebral autoregulation during the intraoperative period, compared with anesthesia induction. The transient decrease of cerebral autoregulation intraoperatively was observed in both RARP and ORP patients, suggesting that factors other than positioning may have contributed to the impairment of cerebral autoregulation. General anesthesia was maintained with sevoflurane in both surgical groups. Sevoflurane has a dose-dependent cerebral vasodilatory effect [43]. Cerebral vasodilation induced by sevoflurane may have contributed to the reduced autoregulatory function during the intraoperative period observed in our study. COx levels decreased after recovery from anesthesia similar to baseline levels, supporting the assumption of transient anesthetic-induced impairment of cerebral autoregulation.

This study has several strengths and limitations that should be addressed. Different blood pressure measurement techniques were used throughout the perioperative period. Baseline blood pressure before anesthesia induction was measured non-invasively with the oscillometric method, whereas invasive blood pressure monitoring was used from induction to recovery from anesthesia. However, both methods for blood pressure measurement were consistently applied in both groups.

We defined baseline blood pressure based on pre-induction measurements, which may be influenced by anxiety and may not be representative of ambulatory blood pressure in the resting sedentary position.

Arterial CO_2_ is a potent regulator of the cerebral vascular tone [44]. We did not draw routine blood gas analyses but used end-tidal CO_2_ values as a surrogate for arterial partial pressure of CO_2_. Provided that ventilation and perfusion are stable, arterial partial pressure of CO_2_ and end-tidal CO_2_ are highly correlated in mechanically ventilated patients [45]. Moreover, a high correlation between arterial and end-tidal CO_2_ has been shown during RARP in the Trendelenburg position [46]. By accepting end-tidal CO_2_ values between 32 and 42 mmHg a potential effect of mild to moderate hypercapnia on the cerebral vasculature has to be taken into account.

In order to rule out impaired cerebral autoregulation because of cerebrovascular disease, we did not include patients with a history of stroke, transient ischemic attack, or carotid and/or vertebral artery disease. These strict exclusion criteria limit the generalizability of our results, since patterns of cerebral autoregulation in patients with preexisting cerebrovascular disease may react differently to head-down positioning, capnoperitoneum, and anesthesia.

One strength of this trial is the continuous monitoring of cerebrovascular autoregulation during different episodes throughout the perioperative period, including anesthesia induction, posture changes, and recovery from anesthesia. Rather than assessing patients only in the steep Trendelenburg position, we performed additional monitoring during open surgery in the supine position and compared a homogenous patient population exposed to two surgical techniques for radical prostatectomy.

## Conclusions

Based on these observational data an estimate of the cerebral autoregulation is unaffected by the position in patients undergoing radical prostatectomy. Our findings suggest that particularly older patients with comorbid conditions such as arterial hypertension may be at risk of undercutting autoregulation limits during general anesthesia. Patients at risk might require blood pressure targets substantially higher than 65 mmHg to maintain cerebral perfusion pressure above their individual lower autoregulatory limit and to ensure adequate regulation of cerebral blood flow. The time correlation method for the continuous measurement of cerebral autoregulation may support perioperative care of high-risk patients in the future.

## Electronic supplementary material

Below is the link to the electronic supplementary material.

Supplementary file1 (DOCX 20 kb)

Supplementary file2 (DOCX 15 kb)

Supplementary file3 (DOCX 16 kb)

Supplementary file4 (DOCX 15 kb)

## Data Availability

The data that support the findings of this study are available from the corresponding author upon reasonable request.

## References

[1] Mottet N, Bellmunt J, Bolla M, Briers E, Cumberbatch MG, De Santis M, Fossati N, Gross T, Henry AM, Joniau S, Lam TB, Mason MD, Matveev VB, Moldovan PC, van den Bergh RCN, Van den Broeck T, van der Poel HG, van der Kwast TH, Rouvière O, Schoots IG, Wiegel T, Cornford P (2017). EAU-ESTRO-SIOG guidelines on prostate cancer. part 1: screening, diagnosis, and local treatment with curative intent. Eur Urol.

[2] Binder J, Kramer W (2001). Robotically-assisted laparoscopic radical prostatectomy. BJU Int.

[3] Haese A, Knipper S, Isbarn H, Heinzer H, Tilki D, Salomon G, Michl U, Steuber T, Budäus L, Maurer T, Tennstedt P, Huland H, Graefen M (2019). A comparative study of robot-assisted and open radical prostatectomy in 10 790 men treated by highly trained surgeons for both procedures. BJU Int.

[4] Ilic D, Evans SM, Allan CA, Jung JH, Murphy D, Frydenberg M (2018). Laparoscopic and robot-assisted vs open radical prostatectomy for the treatment of localized prostate cancer: a Cochrane systematic review. BJU Int.

[5] Pompe RS, Beyer B, Haese A, Preisser F, Michl U, Steuber T, Graefen M, Huland H, Karakiewicz PI, Tilki D (2018). Postoperative complications of contemporary open and robot-assisted laparoscopic radical prostatectomy using standardised reporting systems. BJU Int.

[6] Coughlin GD, Yaxley JW, Chambers SK, Occhipinti S, Samaratunga H, Zajdlewicz L, Teloken P, Dunglison N, Williams S, Lavin MF, Gardiner RA (2018). Robot-assisted laparoscopic prostatectomy versus open radical retropubic prostatectomy: 24-month outcomes from a randomised controlled study. Lancet Oncol.

[7] Herlemann A, Cowan JE, Carroll PR, Cooperberg MR (2017). Community-based outcomes of open versus robot-assisted radical prostatectomy. Eur Urol.

[8] Wallerstedt A, Tyritzis SI, Thorsteinsdottir T, Carlsson S, Stranne J, Gustafsson O, Hugosson J, Bjartell A, Wilderäng U, Wiklund NP, Steineck G, Haglind E, LAPPRO steering committee (2015). Short-term results after robot-assisted laparoscopic radical prostatectomy compared to open radical prostatectomy. Eur Urol.

[9] Yaxley JW, Coughlin GD, Chambers SK, Occhipinti S, Samaratunga H, Zajdlewicz L, Dunglison N, Carter R, Williams S, Payton DJ, Perry-Keene J (2016). Robot-assisted laparoscopic prostatectomy versus open radical retropubic prostatectomy: early outcomes from a randomised controlled phase 3 study. The Lancet.

[10] Nossiter J, Sujenthiran A, Charman SC, Cathcart PJ, Aggarwal A, Payne H, Clarke NW, van der Meulen J (2018). Robot-assisted radical prostatectomy vs laparoscopic and open retropubic radical prostatectomy: functional outcomes 18 months after diagnosis from a national cohort study in England. Br J Cancer.

[11] Forsmark A, Gehrman J, Angenete E, Bjartell A, Björholt I, Carlsson S, Hugosson J, Marlow T, Stinesen-Kollberg K, Stranne J, Wallerstedt A, Wiklund P, Wilderäng U, Haglind E (2018). Health economic analysis of open and robot-assisted laparoscopic surgery for prostate cancer within the prospective multicentre LAPPRO trial. Eur Urol.

[12] Schroeck FR, Jacobs BL, Bhayani SB, Nguyen PL, Penson D, Hu J (2017). Cost of new technologies in prostate cancer treatment: systematic review of costs and cost effectiveness of robotic-assisted laparoscopic prostatectomy, intensity-modulated radiotherapy, and proton beam therapy. Eur Urol.

[13] Awad H, Walker CM, Shaikh M, Dimitrova GT, Abaza R, O'Hara J (2012). Anesthetic considerations for robotic prostatectomy: a review of the literature. J Clin Anesth.

[14] Gainsburg DM (2012). Anesthetic concerns for robotic-assisted laparoscopic radical prostatectomy. Miner Anestesiol.

[15] Kilic OF, Börgers A, Köhne W, Musch M, Kröpfl D, Groeben H (2015). Effects of steep trendelenburg position for robotic-assisted prostatectomies on intra- and extrathoracic airways in patients with or without chronic obstructive pulmonary disease. Br J Anaesth.

[16] Robba C, Cardim D, Donnelly J, Bertuccio A, Bacigaluppi S, Bragazzi N, Cabella B, Liu X, Matta B, Lattuada M, Czosnyka M (2016). Effects of pneumoperitoneum and Trendelenburg position on intracranial pressure assessed using different non-invasive methods. Br J Anaesth.

[17] Blecha S, Harth M, Schlachetzki F, Zeman F, Blecha C, Flora P, Burger M, Denzinger S, Graf BM, Helbig H, Pawlik MT (2017). Changes in intraocular pressure and optic nerve sheath diameter in patients undergoing robotic-assisted laparoscopic prostatectomy in steep 45° Trendelenburg position. BMC Anesthesiol.

[18] Whiteley JR, Taylor J, Henry M, Epperson TI, Hand WR (2015). Detection of elevated intracranial pressure in robot-assisted laparoscopic radical prostatectomy using ultrasonography of optic nerve sheath diameter. J Neurosurg Anesthesiol.

[19] Yoo Y-C, Shin S, Choi EK, Kim CY, Choi YD, Bai S-J (2014). Increase in intraocular pressure is less with propofol than with sevoflurane during laparoscopic surgery in the steep Trendelenburg position. Can J Anaesth.

[20] Mizrahi H, Hugkulstone CE, Vyakarnam P, Parker MC (2011). Bilateral ischaemic optic neuropathy following laparoscopic proctocolectomy: a case report. Ann R Coll Surg Engl.

[21] Weber ED, Colyer MH, Lesser RL, Subramanian PS (2007). Posterior ischemic optic neuropathy after minimally invasive prostatectomy. J Neuroophthalmol.

[22] de Lima-Oliveira M, Salinet ASM, Nogueira RC, de Azevedo DS, Paiva WS, Teixeira MJ, Bor-Seng-Shu E (2018). Intracranial hypertension and cerebral autoregulation: a systematic review and meta-analysis. World Neurosurg.

[23] Lassen NA (1959). Cerebral blood flow and oxygen consumption in man. Physiol Rev.

[24] Donnelly J, Budohoski KP, Smielewski P, Czosnyka M (2016). Regulation of the cerebral circulation: bedside assessment and clinical implications. Crit Care.

[25] Kalmar AF, Dewaele F, Foubert L, Hendrickx JF, Heeremans EH, Struys MMRF, Absalom A (2012). Cerebral haemodynamic physiology during steep trendelenburg position and CO2 pneumoperitoneum. BJA Br J Anaesth.

[26] Schramm P, Treiber AH, Berres M, Pestel G, Engelhard K, Werner C, Closhen D (2014). Time course of cerebrovascular autoregulation during extreme Trendelenburg position for robotic-assisted prostatic surgery. Anaesthesia.

[27] Tsoi KKF, Chan JYC, Hirai HW, Wong SYS, Kwok TCY (2015). Cognitive tests to detect dementia: a systematic review and meta-analysis. JAMA Intern Med.

[28] Xiong L, Liu X, Shang T, Smielewski P, Donnelly J, Guo Z-N, Yang Y, Leung T, Czosnyka M, Zhang R, Liu J, Wong KS (2017). Impaired cerebral autoregulation: measurement and application to stroke. J Neurol Neurosurg Psychiatry.

[29] Ono M, Zheng Y, Joshi B, Sigl JC, Hogue CW (2013). Validation of a stand-alone near-infrared spectroscopy system for monitoring cerebral autoregulation during cardiac surgery. Anesth Analg.

[30] Rivera-Lara L, Geocadin R, Zorrilla-Vaca A, Healy R, Radzik BR, Palmisano C, Mirski M, Ziai WC, Hogue C (2017). Validation of near-infrared spectroscopy for monitoring cerebral autoregulation in comatose patients. Neurocrit Care.

[31] Hori D, Brown C, Ono M, Rappold T, Sieber F, Gottschalk A, Neufeld KJ, Gottesman R, Adachi H, Hogue CW (2014). Arterial pressure above the upper cerebral autoregulation limit during cardiopulmonary bypass is associated with postoperative delirium. Br J Anaesth.

[32] Rupp-Montpetit K, Moody ML (2004). Visual loss as a complication of nonophthalmologic surgery: a review of the literature. AANA J.

[33] Kim M-S, Bai S-J, Lee J-R, Choi YD, Kim YJ, Choi SH (2014). Increase in intracranial pressure during carbon dioxide pneumoperitoneum with steep trendelenburg positioning proven by ultrasonographic measurement of optic nerve sheath diameter. J Endourol.

[34] Roth S, Dreixler J, Newman NJ (2018). Haemodilution and head-down tilting induce functional injury in the rat optic nerve: a model for peri-operative ischemic optic neuropathy. Eur J Anaesthesiol.

[35] Zweifel C, Dias C, Smielewski P, Czosnyka M (2014). Continuous time-domain monitoring of cerebral autoregulation in neurocritical care. Med Eng Phys.

[36] Goettel N, Patet C, Rossi A, Burkhart CS, Czosnyka M, Strebel SP, Steiner LA (2016). Monitoring of cerebral blood flow autoregulation in adults undergoing sevoflurane anesthesia: a prospective cohort study of two age groups. J Clin Monit Comput.

[37] Leoni RF, Paiva FF, Henning EC, Nascimento GC, Tannús A, de Araujo DB, Silva AC (2011). Magnetic resonance imaging quantification of regional cerebral blood flow and cerebrovascular reactivity to carbon dioxide in normotensive and hypertensive rats. Neuroimage.

[38] Tryambake D, He J, Firbank MJ, O'Brien JT, Blamire AM, Ford GA (2013). Intensive blood pressure lowering increases cerebral blood flow in older subjects with hypertension. Hypertension.

[39] Oudegeest-Sander MH, van Beek AHEA, Abbink K, Olde Rikkert MGM, Hopman MTE, Claassen JAHR (2014). Assessment of dynamic cerebral autoregulation and cerebrovascular CO2 reactivity in ageing by measurements of cerebral blood flow and cortical oxygenation. Exp Physiol.

[40] Burkhart CS, Rossi A, Dell-Kuster S, Gamberini M, Möckli A, Siegemund M, Czosnyka M, Strebel SP, Steiner LA (2011). Effect of age on intraoperative cerebrovascular autoregulation and near-infrared spectroscopy-derived cerebral oxygenation. Br J Anaesth.

[41] Gao Y, Zhang M, Han Q, Li W, Xin Q, Wang Y, Li Z (2015). Cerebral autoregulation in response to posture change in elderly subjects-assessment by wavelet phase coherence analysis of cerebral tissue oxyhemoglobin concentrations and arterial blood pressure signals. Behav Brain Res.

[42] Dagal A, Lam AM (2009). Cerebral autoregulation and anesthesia. Curr Opin Anaesthesiol.

[43] Conti A, Iacopino DG, Fodale V, Micalizzi S, Penna O, Santamaria LB (2006). Cerebral haemodynamic changes during propofol-remifentanil or sevoflurane anaesthesia: transcranial Doppler study under bispectral index monitoring. BJA Br J Anaesth.

[44] Meng L, Gelb AW (2015). Regulation of cerebral autoregulation by carbon dioxide. Anesthesiology.

[45] Razi E, Moosavi GA, Omidi K, Khakpour Saebi A, Razi A (2012). Correlation of end-tidal carbon dioxide with arterial carbon dioxide in mechanically ventilated patients. Arch Trauma Res.

[46] Kalmar AF, Foubert L, Hendrickx JFA, Mottrie A, Absalom A, Mortier EP, Struys MMRF (2010). Influence of steep trendelenburg position and CO(2) pneumoperitoneum on cardiovascular, cerebrovascular, and respiratory homeostasis during robotic prostatectomy. Br J Anaesth.

