# Supplementary material for: Comparing the effect of positioning on cerebral autoregulation during radical prostatectomy: a prospective observational study
Source: J Clin Monit Comput. 2020 Jun 20;35(4):891–901. doi: 10.1007/s10877-020-00549-0 (PMC8286946; doi:10.1007/s10877-020-00549-0)
Supplement: Supplementary file 1 — Supplementary file1 (DOCX 20 kb) [file 10877_2020_549_MOESM1_ESM.docx]

**Electronic Supplementary Material 1**

**Title**

Comparing the effect of positioning on cerebral autoregulation during radical prostatectomy – a prospective observational study

**Journal**

Journal of Clinical Monitoring and Computing

**Authors**

Stefanie Beck, Haissam Ragab, Dennis Hoop, Aurélie Meßner-Schmitt, Cornelius Rademacher, Ursula Kahl, Franziska von Breunig, Alexander Haese, Markus Graefen, Christian Zöllner, Marlene Fischer

**Corresponding Author**

Marlene Fischer, MD/PhD, University Medical Center Hamburg-Eppendorf, Department of Anesthesiology, Martinistrasse 52, 20246 Hamburg, Germany, Email: mar.fischer@uke.de.

|  | RARP | ORP | *p* value |
| --- | --- | --- | --- |
|  | n=102 | n=81 |  |
| Prostate volume (TRUS), ml | 37 (30-47) | 34 (27-55) | 0.701 |
| Prostate volume (path), ml | 25 (20-35) | 30 (20-40) | 0.097 |
| Tumor volume, ml | 5.6 (2.8-9.7) | 5.6 (2.8-14.5) | 0.641 |
| Gleason score^a^ |  |  | 0.471 |
| 1 | 7 (6.9) | 6 (7.5) |  |
| 2 | 64 (62.7) | 43 (53.8) |  |
| 3 | 20 (19.6) | 17 (21.3) |  |
| 4 | 0 (0.0) | 2 (2.5) |  |
| 5 | 11 (10.8) | 12 (15.0) |  |
| T category |  |  | 0.448 |
| pT2 | 62 (60.8) | 53 (66.3) |  |
| pT3 | 40 (39.2) | 27 (33.8) |  |
| Number of lymph nodes resected | 13 (8-20) | 15 (7-21) | 0.610 |
| N category |  |  | 0.220 |
| 0 | 82 (80.4) | 58 (72.5) |  |
| 1 | 11 (10.8) | 16 (20.0) |  |
| x | 9 (8.8) | 6 (7.5) |  |
| Positive surgical margin | 21 (20.6) | 22 (27.5) | 0.276 |

**Electronic Supplementary Material 1**: TRUS: transrectal ultrasound. Path: pathological assessment. Data are given as n (%) or median (interquartile range) as appropriate. ^a^Gleason grading according to the 2014 definition of the International Society of Urological Pathology. Numbers do not add up to 183, because the prostate gland was not resected in one patient. RARP: Robot-assisted radical prostatectomy. ORP: open retropubic radical prostatectomy.
