# Supplementary material for: Comparing the effect of positioning on cerebral autoregulation during radical prostatectomy: a prospective observational study
Source: J Clin Monit Comput. 2020 Jun 20;35(4):891–901. doi: 10.1007/s10877-020-00549-0 (PMC8286946; doi:10.1007/s10877-020-00549-0)
Supplement: Supplementary file 2 — Supplementary file2 (DOCX 15 kb) [file 10877_2020_549_MOESM2_ESM.docx]

**Electronic Supplementary Material 2**

**Title**

Comparing the effect of positioning on cerebral autoregulation during radical prostatectomy – a prospective observational study

**Journal**

Journal of Clinical Monitoring and Computing

**Authors**

Stefanie Beck, Haissam Ragab, Dennis Hoop, Aurélie Meßner-Schmitt, Cornelius Rademacher, Ursula Kahl, Franziska von Breunig, Alexander Haese, Markus Graefen, Christian Zöllner, Marlene Fischer

**Corresponding Author**

Marlene Fischer, MD/PhD, University Medical Center Hamburg-Eppendorf, Department of Anesthesiology, Martinistrasse 52, 20246 Hamburg, Germany, Email: mar.fischer@uke.de.

| *Robot-assisted radical prostatectomy (n=102)* | | |
| --- | --- | --- |
| induction | before capnoperitoneum | <0.0001* |
| induction | head-down | <0.0001* |
| before capnoperitoneum | head-down | 0.6408 |
| before capnoperitoneum | PACU | 0.0003* |
| head-down | PACU | <0.0001* |
| induction | PACU | 0.8270 |
| *Open retropubic radical prostatectomy (n=81)* | | |
| induction | intraoperative | <0.0001* |
| induction | PACU | 0.0041* |
| intraoperative | PACU | <0.0001* |

**Electronic Supplementary Material 2**: Comparison of the cerebral oxygenation index between different perioperative episodes using the Wilcoxon signed rank test. *Statistically significant after Bonferroni correction for multiple comparisons. PACU: post-anesthesia care unit.
