# Supplementary material for: Comparing the effect of positioning on cerebral autoregulation during radical prostatectomy: a prospective observational study
Source: J Clin Monit Comput. 2020 Jun 20;35(4):891–901. doi: 10.1007/s10877-020-00549-0 (PMC8286946; doi:10.1007/s10877-020-00549-0)
Supplement: Supplementary file 3 — Supplementary file3 (DOCX 16 kb) [file 10877_2020_549_MOESM3_ESM.docx]

**Electronic Supplementary Material 3a and 3b**

**Title**

Comparing the effect of positioning on cerebral autoregulation during radical prostatectomy – a prospective observational study

**Journal**

Journal of Clinical Monitoring and Computing

**Authors**

Stefanie Beck, Haissam Ragab, Dennis Hoop, Aurélie Meßner-Schmitt, Cornelius Rademacher, Ursula Kahl, Franziska von Breunig, Alexander Haese, Markus Graefen, Christian Zöllner, Marlene Fischer

**Corresponding Author**

Marlene Fischer, MD/PhD, University Medical Center Hamburg-Eppendorf, Department of Anesthesiology, Martinistrasse 52, 20246 Hamburg, Germany, Email: mar.fischer@uke.de.

|  | no arterial hypertension  (n=89) | arterial hypertension  (n=94) | *p* |
| --- | --- | --- | --- |
| *RARP + ORP* |  |  |  |
| COx (induction) | 0.07 (-0.13;0.21) | 0.03 (-0.13;0.16) | 0.207 |
| COx (intraoperative) | 0.21 (0.12;0.28) | 0.19 (0.11;0.3) | 0.796 |
| COx (PACU) | 0.07 (-0.01;0.14) | 0.07 (0.02;0.14) | 0.756 |
| COx (after norepinephrine bolus) | 0.14 (0.01;0.31) | 0.14 (0.00;0.26) | 0.387 |
| *RARP* |  |  |  |
| COx (before capnoperitoneum) | 0.19 (0.04;0.45) | 0.26 (-0.13;0.41) | 0.469 |
| COx (20 min after capnoperitoneum) | 0.15 (0.00;0.25) | 0.18 (0.04;0.32) | 0.550 |
| COx (head-down) | 0.17 (0.11;0.27) | 0.17 (0.11;0.3) | 0.756 |

|  | RARP  (n=53) | ORP  (n=41) | *p* |
| --- | --- | --- | --- |
| COx (induction) | 0.02 (-0.13;0.16) | 0.03 (-0.14;0.10) | 0.897 |
| COx (intraoperative) | 0.18 (0.12;0.29) | 0.25 (0.10;0.32) | 0.515 |
| COx (PACU) | 0.07 (0.01;0.12) | 0.07 (0.02;0.15) | 0.626 |
| COx (after norepinephrine bolus) | 0.15 (0.00;0.31) | 0.13 (-0.04;0.22) | 0.512 |

**Electronic Supplementary Material 3a and 3b**: Median Cerebral Oxygenation Index (COx) with interquartile range in patients with arterial hypertension. Data were compared between patients with and without a history of arterial hypertension (3a). In patients with arterial hypertension (n=94) COx levels were compared between robot-assisted radical prostatectomy (RARP) und open retropubic radical prostatectomy (ORP) using the Mann-Whitney-U test. PACU: post-anesthesia care unit.
