# Supplementary material for: Comparing the effect of positioning on cerebral autoregulation during radical prostatectomy: a prospective observational study
Source: J Clin Monit Comput. 2020 Jun 20;35(4):891–901. doi: 10.1007/s10877-020-00549-0 (PMC8286946; doi:10.1007/s10877-020-00549-0)
Supplement: Supplementary file 4 — Supplementary file4 (DOCX 15 kb) [file 10877_2020_549_MOESM4_ESM.docx]

**Electronic Supplementary Material 4**

**Title**

Comparing the effect of positioning on cerebral autoregulation during radical prostatectomy – a prospective observational study

**Journal**

Journal of Clinical Monitoring and Computing

**Authors**

Stefanie Beck, Haissam Ragab, Dennis Hoop, Aurélie Meßner-Schmitt, Cornelius Rademacher, Ursula Kahl, Franziska von Breunig, Alexander Haese, Markus Graefen, Christian Zöllner, Marlene Fischer

**Corresponding Author**

Marlene Fischer, MD/PhD, University Medical Center Hamburg-Eppendorf, Department of Anesthesiology, Martinistrasse 52, 20246 Hamburg, Germany, Email: [mar.fischer@uke.de](mailto:mar.fischer@uke.de).

**Sample size calculation**

There are no data on the cerebral oxygenation COx in patients with open and/or robot-assisted radical prostatectomy (RARP). One observational study investigated cerebral autoregulation based on measurement of cerebral blood flow velocity using transcranial Doppler sonography in 23 patients during RARP in Trendelenburg position {Schramm:2014hu}. They calculated the cerebral autoregulation index Mx, which was -0.023 in supine position and 0.144 after 25 min in Trendelenburg position. With a standard standard deviation of 0.39, a power of 0.9 and a significance level of 0.05 a sample size of 59 patients would be required to detect a significant difference between supine and Trendelenburg positions using a two-tailed statistical test. We calculated with a drop-out rate of 30%, taking into account technical problems, artifacts, incomplete data export, inability to measure invasive blood pressure or withdrawal of consent.
